# Microcystins in Water: Detection, Microbial Degradation Strategies, and Mechanisms

**DOI:** 10.3390/ijerph192013175

**Published:** 2022-10-13

**Authors:** Qianqian He, Weijun Wang, Qianqian Xu, Zhimin Liu, Junhui Teng, Hai Yan, Xiaolu Liu

**Affiliations:** School of Chemical and Biological Engineering, University of Science and Technology Beijing, Beijing 100083, China

**Keywords:** microcystins, detection, biodegradation, microorganism

## Abstract

Microcystins are secondary metabolites produced by some cyanobacteria, a class of cyclic heptapeptide toxins that are stable in the environment. Microcystins can create a variety of adverse health effects in humans, animals, and plants through contaminated water. Effective methods to degrade them are required. Microorganisms are considered to be a promising method to degrade microcystins due to their high efficiency, low cost, and environmental friendliness. This review focuses on perspectives on the frontiers of microcystin biodegradation. It has been reported that bacteria and fungi play an important contribution to degradation. Analysis of the biodegradation mechanism and pathway is an important part of the research. Microcystin biodegradation has been extensively studied in the existing research. This review provides an overview of (1) pollution assessment strategies and hazards of microcystins in water bodies and (2) the important contributions of various bacteria and fungi in the biodegradation of microcystins and their degradation mechanisms, including mlr gene-induced (gene cluster expressing microcystinase) degradation. The application of biodegradable technology still needs development. Further, a robust regulatory oversight is required to monitor and minimize MC contamination. This review aims to provide more references regarding the detection and removal of microcystins in aqueous environments and to promote the application of biodegradation techniques for the purification of microcystin-contaminated water.

## 1. Introduction

As the population increases, agricultural and urban development are placed under enormous pressure. Eutrophication is caused by the excessive input of exogenous nutrients. With the increasing impacts of climate change and eutrophication, cyanobacterial blooms are one of the global water environment problems, and the environmental pollution and health problems they cause have captured the attention of scientists in various fields. The genera of cyanobacteria that produce cyanobacterial blooms include *Microcystis*, *Planktothrix*, *Nodularia*, *Dolichospermum*, and *Oscillatoria* [1]. These algal outbreaks secrete toxic secondary metabolites, cyanobacterial toxins, of which microcystins (MCs) are the most widely distributed and toxic type of cyanobacterial toxins in cyanobacterial bloom pollution. Potentially toxic cyanobacteria are also increasingly being detected in water supply systems worldwide. In the United States and Canada [2], approximately 80% of water sources and treated water samples contain MCs, with the primary congener being microcystin-LR. Similar investigations from Europe report that MCs account for 60% of the cyanotoxins detected in freshwater [3]. In some regions of Europe, this number reached even higher: MCs were also found in more than 90% of the samples surveyed in a water quality survey of lakes in Greece [4].MCs have also been detected in terrestrial environments irrigated or flooded with water containing harmful algal blooms.

MCs are a group of cyclic heptapeptide toxins with unique amino acid side chains produced by *Microcystis*, *Cichlidium*, *Oscillatoria, Anabaenopsis, Aphanocapsa*, etc. [5]. The general structure of MCs is Cyclo-(-D-Ala-L-X-D-MEASP-L-Z-ADDA-D-Glu-MDHA). There are two important amino acids in MCs that are highly variable, X and Z, which provide the molecular diversity of MCs. The L-X amino acid residue is usually leucine or phenylalanine, while the L-Z amino acid residue is usually arginine, valine, leucine, methionine, or alanine. The structure of ADDA is 3-amino-9-methoxy-2,6,8-trimethyl-10-phenyldeca-4,6-dienoicacid, which is required for the expression of toxin activity. The cyclic structure of MCs makes them resistant to extreme pH, high temperatures, and sunlight. Hughes et al. identified the first MC in 1957, and over 270 variants have since been isolated [6], with varying degrees of toxicity. As far as toxicity is concerned, microcystin-LR (MC-LR) is the most common and most toxic isomer, with microcystin-LA (MC-LA) having equal toxicity to it, followed by microcystin-RR (MC-RR) and microcystin-YR (MC-YR) [7]. Human health can be affected by MCs mainly through physical contact, inhalation, and contaminated water and food. In order to reduce the health risks associated with MCs, the World Health Organization (WHO) recommends that the MC content in drinking water should not exceed 1 μg/L [8].

MCs can be toxic to plants, animals, and even humans. For plants, MCs adversely affect physiological processes, including tissue development, enzyme activation, gene expression, photosynthesis, and other processes necessary for a plant to grow. Additionally, MCs can inhibit or promote mitosis at different doses. Under certain conditions, MCs induce a lag in chromosome formation during mitosis and block early mitosis [9]. It was demonstrated that MCs affect plant cells by disrupting cell microtubules and cytoskeleton. In MC-LR-treated meristematic and differentiated root cells, F-actin exhibited time-dependent alterations that eventually led to the collapse of the F-actin network [10]. Disorganization and eventual depolymerization of microtubules, as well as abnormal chromatin condensation, were observed in the experiments. The disruption of the phytoskeleton to affect the plant was also demonstrated in experiments in which microcystins treated Oryza sativa (rice) root cells [11]. MCs enter the root systems of plants through water in the soil. As a result of MCs’ stability in aqueous environments and the cyclic structure of plants, MCs have persistence of approximately 56 days [3]. Through the root system, MCs are absorbed and then transferred to leaves, shoots, flowers, and fruit tissues. There is the potential for toxin accumulation in edible parts of plants, which can affect the health of humans and animals. A major mechanism of MC toxicity is the inhibition of protein phosphatase PP1 and protein phosphatase PP2A. The hepatotoxicity of MCs is well-established in both animals and humans, but there is also substantial evidence that MCs have effects on the intestine [12], lungs [13], heart [14], brain [15], testes [16], and kidneys [17]. Through the digestive tract, MCs can enter the body via water or by animals and plants that have been contaminated with MCs. As a result, diarrhea, neuroparalysis, liver damage, and in severe cases, poisoning and even death can occur. The use of MC-contaminated water in a hemodialysis unit in Brazil resulted in the death of at least 50 patients in 1998 [18]. As demonstrated by Feng et al., low-dose exposures to MC-LR increased the proliferation of human hepatocellular carcinoma cells [19]. Human intestinal epithelial cell lines are affected by MC-LR through a complex sequence of events, including the inhibition of PP2A activity, the activation of the P53 protein, and the production of reactive oxygen species. Neuronal PP1 and PP2a mRNA levels are inhibited by MCs, leading to neurotoxicity and negative effects, such as learning and memory dysfunction, anxiety, depression, headaches, nausea, inflammation, and neurodegenerative diseases.

**Figure 1:**
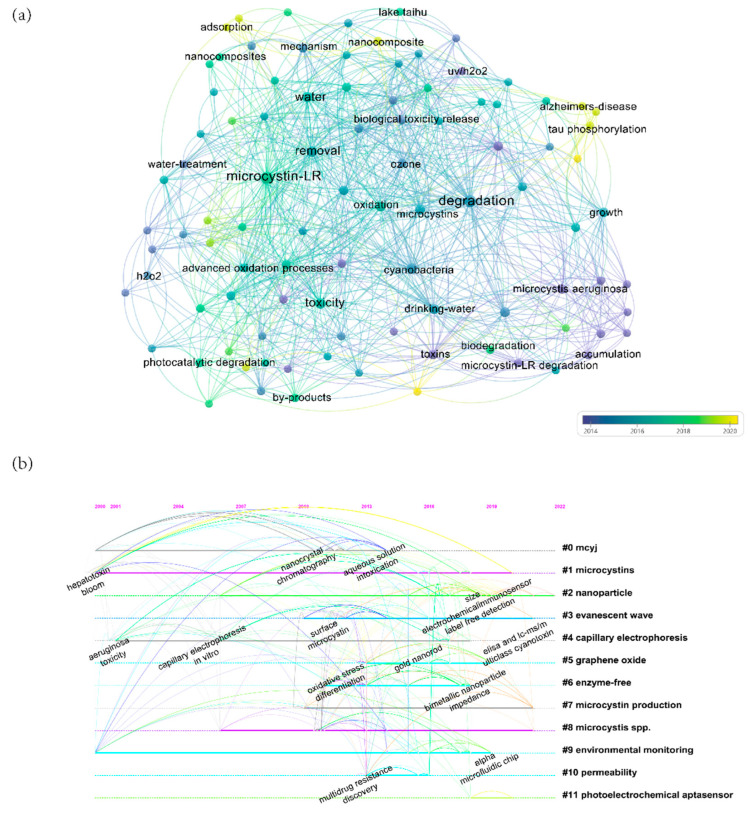
(**a**) Bibliometric map of microcystin degradation studies as visualized from 1063 articles retrieved from the Web of Science database, which were published from 1991 to 2022; (**b**) Citespace keyword timeline map of microcystin detection studies as visualized from 1003 articles retrieved from the Web of Science database. Timespan: 2000–2022 (slice length = 1); selection criteria: g-index (k = 25), LRF = 3.0, L/N = 10, LBY = 5, e = 1.0; network: N = 221, E = 880 (density = 0.0362); largest CC: 213 (96%); nodes labeled: 1.0%; pruning: none; modularity: Q = 0.695; weighted mean silhouette: S = 0.8778; harmonic mean: (Q, S) = 0.7757.

As shown in Figure 1a, the toxicity of microcystins and their accumulation in water have been known since 1982. In recent years, water treatment has attracted attention, and some methods have emerged in the exploration of methods to degrade microcystins, including photocatalytic degradation, advanced oxidation degradation, and biodegradation, among which the biodegradation of microcystins has been particularly important. It has been demonstrated that traditional drinking water treatments (such as coagulation, flocculation, and filtration) can efficiently remove cyanotoxins from cyanobacterial cells and intracellular cyanobacteria, but additional treatment methods are required for extracellular toxins. Several processes have been proposed for natural reductions in microcystin levels, including dilution, adsorption, thermal decomposition aided by pH, photolysis, and biological degradation [20]. However, some processes have limitations in terms of their effectiveness, including limited light penetration for direct photolysis and weak sediment adsorption. In advanced oxidation technology (AOT), titanium dioxide (TiO_2_) was shown to have different efficiencies in the degradation of microcystins by its nanoparticles under UV and visible light, but its degradation performance was affected by pH [21]. Conventional treatment methods remove most cyanobacterial cells. During the coagulation–precipitation process, pre-oxidation can enhance the removal of cyanobacterial cells; however, pre-oxidation may lead to cyanobacterial cell damage (decreased cell viability) and the release of intracellular cyanobacterial toxins, resulting in increased cyanobacterial toxin levels. Since microorganisms can degrade MCs with high efficiency, low cost, and environmental friendliness, they are considered a promising method. As far as removing MCs from aquatic environments is concerned, bioremediation appears to have the greatest potential. MC biodegradation in water bodies has been extensively studied [22].

This article reviews current knowledge regarding the toxic effects and mechanisms of MCs, as well as the progress of research on biodegradation. Finally, the article discusses future opportunities and challenges associated with the biodegradation of MCs.

## 2. Toxic Mechanism of Microcystins

Protein phosphorylation–dephosphorylation is catalyzed by phosphatases and kinases and is an important pathway for regulating protein activity in cells. In eukaryotes, serine/threonine protein phosphatases PP1 and PP2A are critical protein phosphatases [23], and MCs inhibit the action of phosphatases by binding to the catalytic subunits of PP1 and PP2A [24,25]. This process is roughly divided into two steps: first, transient interaction of the toxin with the enzyme, which induces enzyme inactivation, followed by the formation of covalent adducts during the reaction time of several hours after binding, which completely inactivates the enzyme [26]. The softer ADDA side chain is considered to play a key role in this binding process [27]. ADDA is hydrophobic and interacts with four amino acids (Cln122, Ile123, His191, and Trp200) on the catalytic subunit side of PP2A to form a hydrophobic cage [28]. The nuclear phosphoprotein p53, which functions as an activator in DNA repair, apoptosis, and tumor suppression pathways [29], and is a substrate for PP2A and may also be among the proteins regulated by MCs. In mice treated with MC-LR, the p phosphoserine reactivity of the p53 protein was greater in animals treated with MC-LR compared to the saline-treated group. This experiment illustrated that MC-LR induces the hyperphosphorylation of P53 by inhibiting the action of PP2A, leading to apoptosis [30].

The binding of MCs to PP1 and PP2A can, in addition to regulating p53 to affect apoptosis, trigger many negative responses. The expression of mitogen-activated protein kinases (MAPKs) is mediated by PP2A, which regulates the expression of proto-oncogenes, the latter being involved in the transcription of genes for growth and differentiation. bcl-2 acts as a homodimer protecting the integrity of mitochondria and is a crucial cell death regulator; however, it is a direct substrate of PP2A [31]. MCs affect the expression of such related proteins through the inhibition of PP2A [32], resulting in reduced cell function or even apoptosis.

## 3. Detection

In order to manage and control MCs in a targeted manner and to prevent or reduce the health hazards associated with MCs, sensitive, rapid, and reliable detection methods are urgently needed. Figure 1b clearly illustrates the development of MC detection techniques. The classical detection techniques, enzyme-linked immunosorbent assays (ELISAs) and liquid chromatography-mass spectrometry (LC-MS) are still widely employed, as are biosensors and microarrays, which contribute to environmental monitoring. More efficient separation techniques are also being developed to improve detection efficiency. Various methods have been developed, such as protein phosphatase inhibition assays (PPIA), ELISAs, high-performance liquid chromatography (HPLC), LC-MS, and biosensors [33,34]. Chemical methods, such as HPLC and LC-MS, can identify various variants more sensitively, but they are expensive, the equipment is large in size, expertise is needed to use them correctly, making it difficult to promote their application in actual water monitoring, while PPIAs, ELISAs, and other biological techniques are more simple and specific. In terms of portability, the advantages of biosensors are more prominent. Emerging methods, such as biosensors, which are easy to detect in practice because of their rapidity and portability, provide new ideas for the detection of MCs.

### 3.1. Liquid Chromatography-Mass Spectrometry (LC-MS)

Liquid chromatography-mass spectrometry (LC-MS) is a classical technique applicable to the determination of the content of environmental substances. This technique was first applied to the structural determination of MCs by Bishop et al. in 1959 [35]. However, due to the limitations of the technology at that time, the detection of the complete structure was not achieved until 1984, when, with the development and application of mass spectrometry, Dawie P. Botes completed the detection of the complete structure of the cyclic heptapeptide toxin cyanoginosin-LA [36]. Currently, this technique has been developed, and several LC-MS techniques have contributed to the detection of MCs [37,38].

By combining the separation capacity of chromatography with the high sensitivity and selectivity of mass spectrometry, LC-MS reflects the complementary advantages of chromatography and mass spectrometry, resulting in high separation ability for complex samples. It is capable of revealing relative molecular mass and structural information, as well as enabling high-throughput detection of MCs [39]. One of the more effective and efficient methods is LC/TOF-MS. Compared to other quadrupole mass spectrometers, a TOF mass spectrometer provides greater signal resolution on the m–z axis. It is able to collect data over a large mass range without any reduction in sensitivity, thus achieving full spectral sensitivity and resolving the possibility of interference unrelated to the signal of interest with high resolution [40]. It is more accurate, has a higher method specificity, and is capable of rapidly and efficiently detecting MC variants [41,42,43]. According to Pekar’s study, he utilized this technique to simultaneously identify 22 different variants of MC [44]. According to Turner et al., they developed and optimized a new ultra-high-performance liquid chromatography-tandem mass spectrometry method for the simple and rapid determination of MCs in a wide range of matrices that did not require complex pre-analytical concentration procedures [45].

Solid-phase extraction (SPE) can improve the recovery of analytes. The separation of analytes from interfering components is more effective, and sample pretreatment is reduced. SPE was shown to be applicable to water quality detection [46], but the downside is that some SPE cartridges can be expensive, and high cost is not conducive to the promotion of technology. An improved method was developed by Fayad using solid-phase extraction, liquid chromatography, and tandem mass spectrometry (LC-MS/MS) for the rapid detection of MCs in water samples, despite the influence of the sample matrix [47].

LC-MS can detect MCs in water sources at an early stage, but its high sensitivity and selective specificity require specialized equipment and personnel operation and are limited by the cost of the equipment, which makes it inconvenient to apply for field detection.

### 3.2. Enzyme-Linked Immunosorbent Assay (ELISA)

An ELISA is based on the interaction of MC-LR and NODS with monoclonal antibodies (MAbs) or polyclonal antibodies (PAbs) produced against them. Such Abs are detected by the recognition of the unusual amino acid ADDA, which becomes a common recognition site. It was the discovery of antibodies against ADDA in MC variants that led to the development of ELISAs. The first anti-MC polyclonal antibody was discovered by Brooks et al. in 1988, but it was not successfully applied to an assay [48]. The next year, Chu et al. successfully applied this technique to the detection of MC levels in animals [49]. Since then, this technique has been continuously developed. In addition to the new ELISA method for immunizing chicken egg antibodies, the competitive indirect ELISA (CI-ELISA) method for sheep anti-6(E)ADDA antibodies, and the competitive direct ELISA (CD-ELISA) method, all of these methods are capable of detecting water samples and reducing detection concentrations to low levels [50,51,52]. For routine determinations of MC content, an indirect competitive enzyme-linked immunosorbent assay (ELISA) based on group-specific monoclonal antibodies was developed, considering the need to target MC variants to the maximum extent possible [53].

With the advantages of high specificity, high sensitivity, and fast detection, ELISAs have become a widely used screening technique that can detect relative changes in the concentrations of MCs in water bodies, which is important for water management. Comparable to other methods, ELISA results are easily reproducible [38,54]. The detection concentration is lower than the WHO standard value and is often used for the testing of water quality in real-life situations. There are several advantages to this kit, including ease of operation in the field, instant detection, rapid screening, etc. The structure generally resembles that of a 96-well plate [55,56].

The limitations of ELISAs are high cost of equipment, time-consuming analysis, and high requirements for personnel operation. Additionally, although an ELISA can identify different MC variants, it cannot evaluate the toxicity of MC variants due to variations in antibody specificity [50,51,52], so it cannot be applied as a standard analytical method for identifying MCs. Therefore, ELISAs and LC-MS are commonly used in conjunction to conduct detection.

### 3.3. Protein Phosphatase Inhibition Assay (PPIA)

A PPIA is an enzymatic assay that was established on the condition that MCs are specific inhibitors of PP1 and PP2 [5,25]. MCs specifically bind and inhibit the activity of serine and threonine phosphatases, such as PP1 and PP2a. PPIAs detect the amounts of MCs by measuring changes in the amounts of phosphate released from phosphorylated protein substrates. The first PPIA was used in 1994 when J An et al. combined a colorimetric protein phosphatase inhibition assay with an enzyme-linked immunosorbent assay to study microcystins and nodulin [57]. Various other PPIA techniques have been gradually established since then, including the variance colorimetric immuno-PPIA (CI-PPIA) [58] in combination with immunoassays and immunocapture PPIA (IC-PPIA) [59], using the antibody-specific separation of MCs. In an experiment to detect different MC variants in reservoirs and artificial ponds in Okinawa, Japan, Tsuyoshi Ikehara et al. applied a PPIA to identify and isolate Microcystis aeruginosa strains producing five MC variants (MC-LR, -RRR, -LA, -FR, and -WR), which was the first report of Microcystis aeruginosa strains producing mainly MC-WR and -FR toxins in Japan [60]. In addition, the method is also applicable to the detection of other protein phosphatase inhibitors, such as okadaic acid and mutamycin [61].

A PPIA, which usually needs to be performed on a 96-well microtiter plate [62], is a simple, convenient, economical, and rapid method for MC detection with high sensitivity and is suitable for testing a large number of samples as a routine detection technique. However, due to its tendency to overestimate toxin concentrations and lack of specificity for MC variants [56,63], this method can only be used as a screening method. In this respect, PPIAs can meet the requirement of significantly reducing the number of unnecessary samples to be analyzed [64].

### 3.4. Biosensor Methods

Traditionally, methods such as enzyme-linked immunosorbent assays (ELISAs) and liquid chromatography-mass spectrometry (LC-MS) are the most commonly used techniques for the detection and quantification of cyanotoxins. However, these techniques usually have disadvantages, such as expensive equipment, time-consuming procedures, and difficulty of detection in the field. Considering these reasons, biosensors have become a complement to traditional detection and are gradually becoming a global focus as a new monitoring tool [65]. Biosensors are analytical tools consisting of a biometric element bioreceptor in direct contact with a sensor.

Biosensors are rapidly developing, combining traditional strategies for detecting MCs with sensors, and there are now colorimetric, electrochemical, fluorescent, and plasma biosensors. Among them, electrochemical biosensors, which are miniaturized, portable, and unaffected by colored or opaque substrates [66,67], have attracted much attention. There are electrodes as conversion elements in electrochemical biosensors, as well as immobilization carriers and immobilized biological components on the electrodes to convert target molecules and their reaction signals into electrical signals through specific recognition between biomolecules to achieve the qualitative or quantitative detection of target analytes. Long Feng et al. developed a biosensor for detecting MC-LR using the electrical properties of graphene [68]. Keke Zhang et al. immobilized calf thymus DNA (ctDNA) on an electrode, and the presence of MC-LR affected the conformational change of immobilized ctDNA and reduced the electron transfer impedance, thus enhancing the electrochemical response. The method was reported to have a linear range of 4–512 ng/L and a detection limit of 1.4 ng/L, which is 700-fold lower than the guideline level recommended by the World Health Organization [69]. Nanocomposites have become a popular material for biosensors. Single-walled carbon nanotubes (SWNTs) are able to make paper conductive, and changes in the conductivity of paper can be used to detect MC-LR content [70]. In recent years, aptamer-based biosensor technology has been developed. Aptamers are single-stranded DNA or RNA oligonucleotide or peptide molecules that bind to a specific target and bind to the target to facilitate detection [71]. Eissa et al. designed a sensitive and selective aptamer with a significantly higher peak current in the presence of MC-LR [72].

Due to its simplicity, sensitivity, portability, and ability to analyze in situ, biosensor technology offers a more effective way to monitor MCs quantitatively. However, more efforts are needed to demonstrate the advantages of biosensors in real samples.

## 4. Biodegradation

The possible pathways of microcystin production can be summarized as adsorption, physical and chemical decomposition, bioaccumulation, and biodegradation. Among these, biodegradation in water has been shown to be very effective. Currently, organisms with MC degradation capabilities include prokaryotes (e.g., bacteria) and eukaryotes. These findings provide ideal materials for exploring the basics of microbial degradation and practical applications. It has been discovered that a variety of microorganisms can degrade MCs, providing an important biological means of controlling and managing MC pollution. Numerous researchers have identified indigenous bacteria capable of degrading MCs from eutrophic lakes, ponds, and rivers. These strains include *Sphingomonas* sp., *Novosphingobium* sp., *Stenotrophomonas* sp., *Bacillus* sp., and fungi, with different MC degradation rates.

### 4.1. Bacteria

In 1994, Jones et al. [73] determined that *Sphingomonas* sp. ACM-3962 was able to degrade MCs, considering them as the sole source of carbon and nitrogen to sustain its own growth. This was the first degrading strain, and subsequently, degradation experiments with other strains began. A variety of strains have been reported to have been shown to degrade MCs (Table 1). Most MC-degrading bacteria belong to the genus *Sphingomonas*, and *Acinetobacter, Arthrobacter*, *Bacillus*, *Novosphingobium*, *Paucibacter*, *Pseudomonas*, *Sphingopyxis*, and *Stenotrophomona* have also been shown to some strains with the ability to degrade. Most of these degrading bacteria perform the degradation of MCs under aerobic conditions [74], using them as a carbon source to accomplish the degradation. *Paucibacter sp.* CH [75], a bacterium capable of degrading microcystins, was isolated from the sediments of Chaohu Lake, China, and degraded 11.6 μg/mL of MCL to below the detection limit within 10 h using MC-LR as the sole carbon and energy source. However, the degradation rates of MCs were found to be higher under anaerobic conditions [76]. The degradation potential of the amino acid anaerobic strain ALA-1 for MC-LR was demonstrated by Bao et al. This strain was able to degrade 4 mg/L MC-LR to levels below the detection limit within 10 days at moderate temperatures without a lag period [77]. An analysis of MCs in cyanobacterial cells and the biodegradation of MC-YR, -RR, and -LR by *Sphingomonas* sp. USTB-05 at the cellular and enzymatic levels in Yunnan Dianchi was investigated by Yan et al. The initial 14.8 mg/L of MC-YR was completely eliminated by crude enzymes (CEs) of *Sphingopyxis* sp. USTB-05 within 10 h. This is a promising bacterial strain that we isolated and identified in our previous study [78].

### 4.2. Fungi

In addition to bacteria, some eukaryotes have been found to degrade MCs. In 2012, Zhang and Xie [97] isolated and identified the first fungal strain capable of degrading MCs. In this study, Microcystis aeruginosa-LR (MC-LR) was used as the degrading fungus, and the target was biodegraded by *S. commune*. The complete degradation of MC-LR took 2 d under optimal conditions at an initial mass concentration of 1 mg/L. Currently, about six fungal strains have been identified as MC-degrading fungi [97,98,99,100,101,102]. The principle of the fungal degradation of MCs has been variously described. Studies on the efficiency of MC removal by *Mucor hiemalis* showed that fungal intracellularly internalized MC-LR was broken down and biotransformed into less toxic MC-LR-GSH conjugates within 24 h of exposure [102]. Zeng et al. [103] found that *Pseudomonas flavus* not only inhibited the growth of P. aeruginosa, but its metabolites were able to inhibit the synthesis of MCs.

### 4.3. Molecular Mechanisms

Bourne et al. [104,105] have described the pathways and genes involved in the biodegradation of MC-LR. The degradation of MC-LR is considered to be mediated by at least three intracellular hydrolases, and two intermediates of enzymatic degradation of MC-LR have been identified, namely linearized (acyl-ring) MC-LR (NH2-Adda-Glu-Mdha-Ala-Leu-MeAsp-Arg-OH) and tetrapeptide MC-LR (NH2-Adda-Glu-Mdha-Ala-OH). In the first step, the hydrolysis of MC-LR leading to ring opening is catalyzed by an enzyme called microcystinase, now known as MlrA. The initial site of action of this enzyme is at the ADDA-Arg peptide bond, transforming the cyclic MC-LR into linear MC-LR. The linear MC-LR product is 160-fold less reactive than the parent compound to protein phosphatases, suggesting that the reaction through MlrA-mediated molecular toxicity is greatly reduced. MlrA has previously been characterized as a metalloprotease through inhibition studies. Inhibition studies have likewise characterized the second enzyme in the degradation pathway (formerly peptidase 2, now defined as MlrB) as a possible serine peptidase, with MlrB responsible for further breaking the peptide bond linking alanine to leucine in the linear MC-LR peptide chain to produce the tetrapeptide compound. A third enzyme (formerly peptidase 3, now defined as MlrC) characterized as a possible metallopeptidase is responsible for degrading the tetrapeptide compound even further, with the products being smaller amino acids and peptides. Since then, various studies have designed qualitative polymerase chain reaction (PCR) methods to detect the MlrA, MlrB, and MlrC genes, especially MlrA, which is involved in the cleavage of the microcystin ring structure. MlrD can facilitate the transport of MCs or their biodegradable products across the bacterial cell wall. ADDA is the active product of MlrC on linearized MC-LR; however, there is a hexapeptide (NH2-Glu(6)-Mdha(7)-Ala(1)-Leu(2)-MeAsp(3)) that remains. This hexapeptide cannot be further degraded by MlrC but can be hydrolyzed into unknown products by the MlrA and MlrB of ACM-3962. Our laboratory identified the MlrA active site through molecular dynamics simulations, homology modeling, and docking, and determined that the enzyme belongs to the glutamate protease of type II CAAX isoprenoid endopeptidase. These new findings provide valuable additions to previous studies [69]. Our laboratory recently analyzed the whole genome of *Sphingopyxis* sp. USTB-05 based on second- and third-generation sequencing technologies, and the results expanded our understanding of the pathway of the complete biodegradation of cyanobacterial hepatotoxins by *Sphingopyxis* sp. USTB-05 [106].

In addition, previous studies have elucidated the biodegradation pathways of the MC variants MC-RR and MC-YR. *Sphingopyxis sp.* MB-E was described for the first time [92], and its degradation of MC-RR, -YR, -LY, -LY, -LW, and -LF was observed, while the presence of the above gene clusters was confirmed. Our group used *Sphingopyxis sp.* USTB-05 as an experimental subject and found that the first enzyme, MlrA, converted the cyclic MC-RR or MC-YR into linear form; the second enzyme attacked the Ala\\Arg or Ala\Tyr bond of the linearized MC-RR or MC-YR to produce a tetrapeptide; and finally, the third enzyme, Mlr, further degraded the tetrapeptide to produce ADDA [107,108,109].

## 5. Application and Challenges

In general, MCs can be degraded by microorganisms in water, so the technique of microbial degradation can be used for the removal of microcystins in water. It was found that the degradation efficiency of degrading bacteria was limited when the water body contained high concentrations of MCs [110]. In addition, when multiple colonies coexisted in a natural environment, biological interactions occurred that affected the degradation efficiency of microcystins, most likely due to the acidic metabolites of other coexisting bacteria that hindered the degradation of microcystins by lowering the pH [111]. Using a filtration system, the flow of wastewater is used to keep the concentration of microcystins and the pH of the water column stable, as well as to improve the overall degradation efficiency. The initial filtration system is easy to maintain, but the degradation process is slow under conditions that rely on microbial degradation in the water alone, requiring a long adaptation period and limiting the treatment efficiency. After screening for strains that effectively degrade MCs, the degrading bacteria are immobilized on a membrane system to form a biofilm filtration system that can treat wastewater more efficiently.

In filtration systems, sand is often used as a co-media (or carrier) for building bio-films, but also other filter media can be used, such as glass, plastic, resin, porous ceramics, charcoal, graphene oxide, and reduced graphene oxide [112,113]. A study by Xu et al. [114] found that granular activated carbon bioactive filtration units achieved 54.8–72.0% removal of MC-LR and proposed an efficient treatment strategy. Afterward, considering that the efficacy of biofiltration membrane systems depends largely on the successful attachment of degradative bacilli to the media, GAC was selected as a better filtration medium than sand because of its rough and crack-rich surface, which could protect the newly attached microorganisms to some extent [115]. In the same work for treating wastewater, Awual et al. designed a new organic ligand-embedded macroporous lightweight composite to capture heavy metal ions in water, and the experimental results showed that the addition of the composite had a synergistic effect on the metal adsorption capacity [116]. The material was able to absorb specific metal ions, even in the presence of a large number of coexisting metal ions. This indicated that the composite material plays an important role in capturing contaminants and is specific. They also investigated the use of naturally available wheat flour (WF) carbohydrate polymer biodegradable adsorbents for the effective removal of cationic dyes from contaminated water [117]. Natural biosorbents could be a widely used class of materials to safeguard public health. In addition, the group’s research on functionalized ligands for the immobilization and preparation of adsorbents using environmentally friendly processes and novel chemical sensors for contaminant monitoring is worthwhile [118,119,120,121].

The nature of degrading bacteria also plays a big role in the function of a biofiltra-tion system. In terms of attachment, degrading bacteria need to be screened for good adhesion to the filter media, and in terms of degradation, degrading bacteria need to be screened for applicability in real-world environments. In practical applications, the deg-radation efficiency of degrading bacteria seems to be reduced, which Li and Pan [122] attributed to the fact that degrading bacteria are usually isolated using optimized artificial media with MCs as the only substrate, and there is little competition with other bacteria during the isolation process. Maximum efficacy may not be achieved in a real environment. Therefore, new natural media isolation methods should be used to screen for degrading bacteria that are more suitable for functioning in the water column. A fraction of fungi reduces MCs by inhibiting the growth of toxic cyanobacteria. In biofiltration membrane systems, fungi and degrading bacteria can be co-colonized on top of the filter membrane, which may have better results. However, the effect of the combination of fungi and bacteria on the degradation efficiency of microcystins needs further study, and the application of co-colonization of degrading fungi with degrading bacteria needs more experiments to prove its feasibility.

**Figure 2:**
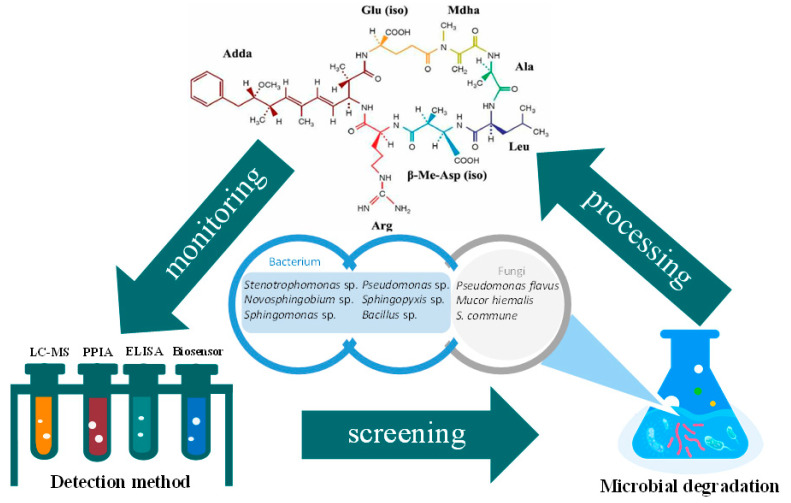
The engineering system of microcystin microbial degradation. It includes a series of processes of screening degrading microorganisms, microbial degradation for water treatment, and real-time monitoring.

As shown in Figure 2, starting from a microcystin-contaminated water body, the degree of change in the pollutants is displayed by the detection method, and the microorganisms that can degrade microcystin are screened out, combined with a biofilm system, and applied in water treatment. At the same time, simple, portable, and sensitive detection methods are used for on-site monitoring. The formation of a systematic treatment process is beneficial to the health of water bodies and ensures public safety.

## 6. Conclusions

Microcystins produced by cyanobacterial blooms can cause serious pollution of the water environment and even endanger human and animal health. Therefore, finding effective methods for the detection and treatment of MCs is essential to maintaining global public health security. After recognizing the hazards of MCs, in terms of detection, it is essential to develop some safe, rapid, and highly sensitive detection methods for early detection and treatment, as high concentrations of MCs are not conducive to degradation efficiency. More practical, simple, and portable detection methods need to be developed urgently, and in recent years, biosensing detection methods have become a hot spot for research due to their sensitivity and portability. In terms of degradation technology, although biodegradation methods have been proved to be relatively effective in experiments, more in-depth research is needed on how to practically apply them in real-life situations and continuously improve water treatment strategies. While continuing to screen degradation bacteria suitable for real-life water environments, it is also important for the continuous innovation of attachment media and adsorbents. Considering that MCs are present in most water bodies, including produced water, the application of environmentally friendly processes and natural materials is a concern in the future.

## Figures and Tables

**Table 1:** Bacteria involved in the degradation of microcystins.

Bacterium	Degradable Analogues	Degrading Genes	References
*Acinetobacter* sp. CMDB-2^c^	MC-LR	Unknown	[79]
*Bacillus flexus* SSZ01	MC-RR	mlrA	[80]
*Bacillus* sp. AMRI-03	MC-RR	mlrA	[80]
*Bacillus* sp. EMB	MC-LR, MC-RR	mlrA	[81]
*Bordetella* sp. MC-LTH1	MC-LR,MC-RR	mlrA	[82]
*Delftia acidovorans* USTB04	MC-LR, MC-RR	Unknown	[83]
*Enterobacter* sp. YF3	MC-LR	Unknown	[84]
*Methylobacillus* sp. *J10*	MC-LR, MC-RR	Unknown	[85]
*Novosphingobium* sp. KKU15	[Dha^7^]MC-LR^f^	Unknown	[86]
*Novosphingobium* sp. KKU25s	[Dha^7^]MC-LR^f^	mlrA–mlrD	[87]
*Novosphingobium* sp. THN-1	MC-LR	mlrA–mlrD	[88]
*Pseudomonas aeruginosa*	MC-LR	Unknown	[89]
*Sphingomonas* sp. ACM-3962	MC-LR, MC-RR	mlrA–mlrD	[73]
*Sphingopyxis* sp. LH21	MC-LR, MC-LA	mlrA–mlrD	[90]
*Sphingomonas* sp. MD-1	MC-LR, MC-RR, MC-YR	mlrA–mlrD	[91]
*Sphingopyxis* sp. MB-E	MC-LR, MC-YR, MC-LF, MC-LY, MC-LW	mlrA–mlrD	[92]
*Sphingopyxis* sp. TT25	MC-LR, MC-RR, MC-YR,MC-LA	mlrA	[93]
*Sphingopyxis* sp. USTB05	MC-LR, MC-RR, MC-YR	mlrA–mlrC	[94]
*Sphingomonas* sp. Y2	MC-LR, MC-RR, MC-YR	mlrA	[95]
*Stenotrophomonas* sp. EMS	MC-LR, MC-RR	mlrA	[96]

## Data Availability

Not applicable.
